# Predictors for viral load suppression among HIV positive adults under ART treatment in University of Gondar Comprehensive Specialized Hospital: retrospective cohort study

**DOI:** 10.1038/s41598-024-53569-0

**Published:** 2024-02-08

**Authors:** Nurye Seid Muhie

**Affiliations:** Department of Statistics, College of Natural and Computational Science, Mekdela Amba University, Tulu Awuliya, Ethiopia

**Keywords:** Diseases, Medical research, Risk factors

## Abstract

HIV continues to be a serious global public health concern, having 40.4 million lives up to now and continuing to spread throughout all countries. The objective of this study was to identify predictors for viral load suppression among HIV positive adults under ART treatment in University of Gondar Comprehensive Specialized Hospital, Ethiopia. An institution based retrospective cohort study design was carry out from 30th March 2017–30th March 2022.Accelerated failure time model were employed to get wide-ranging information about adult HIV positive patients. In this study out of 378 study participants, about 77.8% were suppressed viral load count and the rest were censored. The Weibull AFT model results revealed that predictors were older age (φ = 0.774, 95% CI 0.602–0.793), primary educators (φ = 0.931, 95% CI 0.809–0.964), patients disclosed the disease to family member (φ = 1.093, 95% CI 1.001–1.457), viral load < 10,000 copies/mL (φ = 1.153, 95% CI 1.015–1.309), hemoglobin level ≥ 11g/dL (φ = 1.145, 95% CI 1.028–1.275), CD4 cell count ≥ 200 per mm^3^ (φ = 1.147, 95% CI 1.019–1.290), weight ≥ 50 kg (φ = 1.151, 95% CI 1.033–1.275), BMI between 18.5 and 24.9 kg/m^3^ (φ = 1.143, 95% CI 1.007–1.296), fair treatment adherence (φ = 1.867, 95% CI 1.778–1.967), good treatment adherence (φ = 1.200, 95% CI 1.046–1.377), advanced WHO clinical stages (φ = 0.923, 95% CI 0.899–0.946), patients with OCC (φ = 0.821, 95% CI 0.720–0.936) and substance use (φ = 0.876, 95% CI 0.773–0.993) statistically significant predictors for viral load suppression at 5% level of significance. Then, near intensive care of adult patients’ whose ages between 25 and 34 years, primary educational level, advanced WHO clinical stage, patients with OCC, and substance users can help them improve their health and live longer. Lastly, further studies should be done on HIV positive adult patients by considering other important independent variables that were not included in this study.

## Introduction

HIV continues to be a serious global public health concern, having 40.4 million lives up to now and continuing to spread throughout all countries. By the end of 2022, there were an estimated 39.0 million HIV-positive individuals worldwide, with 25.6 million of them individuals living in Africa. An estimated 660,000 people acquired by HIV in 2022 and 380,000 deaths with HIV-related causes^1^. Ethiopia has also one of the largest HIV infected population among sub-Saharan Africa countries with an estimated 691,362 people were living in 2019^2^.

Antiretroviral therapy (ART) aims to improve the prognosis and quality of life for patients living with HIV^3^ by reducing the rate of disease occurrence, progression, mortality^4^, and morbidity^5^. This successful ART leads to high chance of HIV viral load suppression ^6^ and undetectable levels of virus in both improving individual health and halting onward. Initial viral load testing in people with HIV should be measured after 6 months of initiating ART and every one year thereafter routinely. Patients viral load count less than or equal to 1000 copies per mL, which implies that HIV is controlled by the current ART regiment and also commonly referred to as having a suppressed viral load count^7^.

The 90–90–90 targets were introduced in 2014 by the Joint United Nations and they set a goal of 90% of all HIV-positive individuals being diagnosed, 90% of those being diagnosed receiving ART, and 90% of those receiving treatment achieving viral load suppression by 2020^8^. However, world health organization recommends viral load monitoring to ensure viral load suppression is achieved and maintained, but large gaps in global access remain, particularly in low- and middle-income countries^9^.

Ethiopia adopted the global 90–90–90 target which is part of the strategies designed to eliminate HIV/AIDS epidemics by 2030 and has been working with many collaborators and stakeholders in improving disease detection, viral load testing and adherence on antiretroviral therapy^2^. However, in different studies of Ethiopia viral load suppression is very far from achieving the UNAIDS 90–90–90 target. For instance, the suppression rate of viral loads is 80.9% in Arba Minch and 72% in the eastern Shewa zone, respectively^10,11^.

Numerous studies have been conducted on the clinical and sociodemographic parameters influencing HIV viral load suppression. Among them some of the studies indicated that the predictors, like marital status, baseline CD4 cell count, baseline viral load < 10,000 copies per mL, baseline Cotrimoxazole preventive therapy (CPT), baseline Isoniazid preventive therapy (IPT) and good adherence level to ART^10,11^, Poor treatment adherence^12^, alcohol drinking, non- disclosure of HIV status^13^, and duration of ART^14^ were considered as predictors that affects viral load suppression among adult HIV patients.

Additionally, the current study site is near to Bahir Dar, one of Ethiopia's HIV-positive districts, and a significant number of HIV patients visit in university of Gondar comprehensive specialized hospital. Then, there is scarcity of study have been done by Weibull accelerated failure time (AFT) model among HIV patients in these study area. Consequently, the present study conducted by Weibull AFT model to analysis survival time to viral load suppression. Therefore, the objective of this study was to identify predictors for viral load suppression among HIV positive adults under ART treatment in University of Gondar Comprehensive Specialized Hospital (UGCSH), Ethiopia.

## Material and methods

*Data source* The source of data for this study is secondary data source because the study participant is adult HIV positive patients who registered and under follow-up from 30th March 2017 to 30th March 2022.

*Study design* A retrospective cohort study design was conducted on adult HIV positive patients registered at University of Gondar comprehensive specialized Hospital.

**Figure 1:**
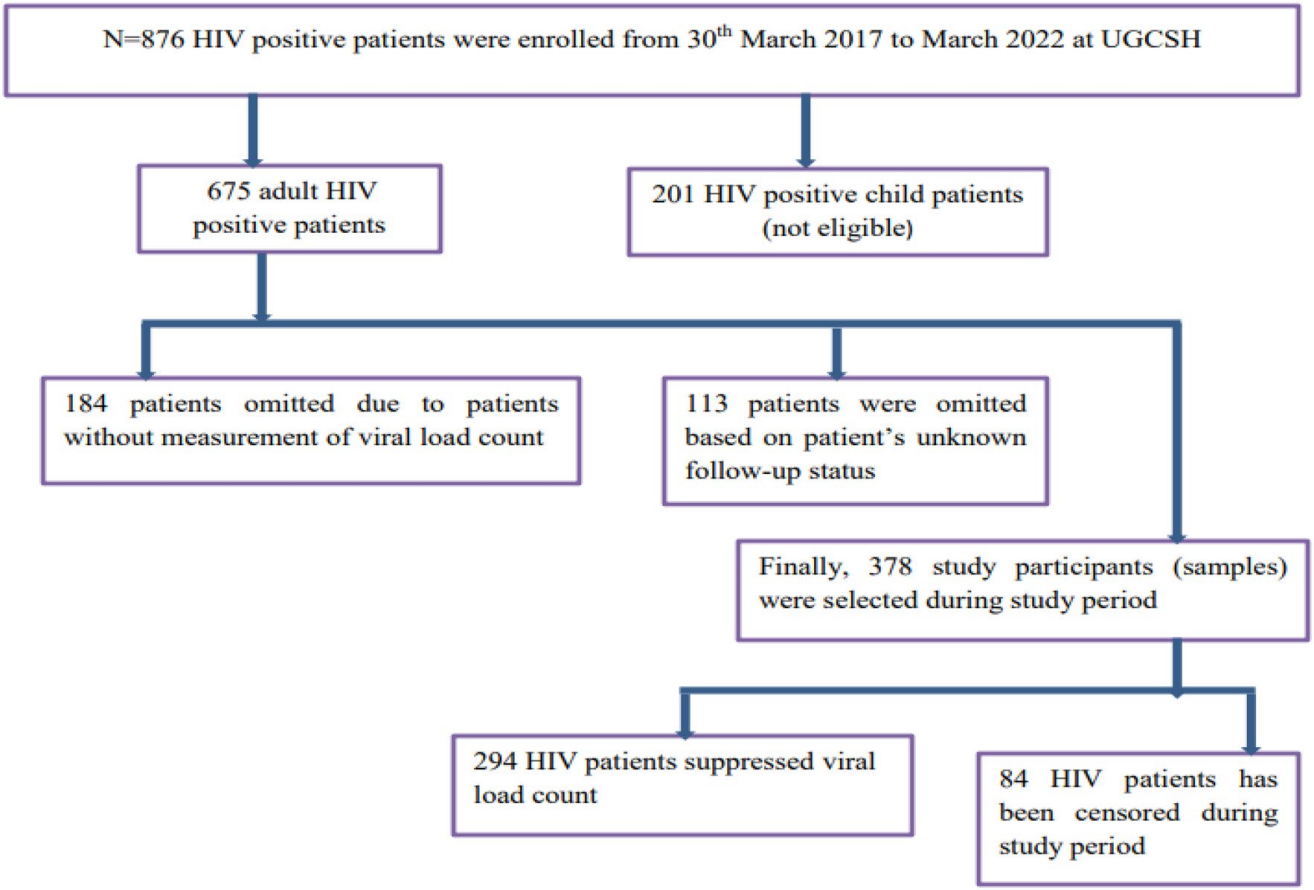
Sample size determination conceptual frame work among adult HIV positive patients.

*Procedure of choosing study participants* Patients didn’t have full recorded information regarding viral load count and unknown patents follow up status were excluded. The survival time of these patients after treatment was determined, and patients transfer out, loss to follow-up, death, and still alive at the end of study time were considered censored. Finally, for this study there are 378 study participants were selected based on the above criteria (Fig. 1).

*Data collection procedure* The data collection procedure was based on patient's chart and electronic database system. Patient charts were selected by using their medical registration number (MRN) of patients and the necessary information (socio demography, behavioral and clinical variable) was retrieved from patient charts by trained ART data clerks.

*Data quality*: In order to ensure the quality of collected data, two day intensive training was given for ART data extractors based on the objective of this study. All extraction processes are overseen by data extractors. During data extraction, ART data management and the principal investigator checked the checklists for completeness and consistency daily.

*Response variable* The response variable for this study was survival time to viral load count suppression of HIV positive patients. In this study, viral load count ≤ 1000 copies per mL can be classified suppressed and viral load count unsuppressed > 1000 copies per mL.

*Independent variables* Gender, age in years, residence, marital status, religion, level of education, disclosure status, and substance use, CD4 cell count per mm^3^**,** baseline viral load count in copies per mL, hemoglobin level in g/dl, weight in kg, body mass index (BMI) in kg per m^3^, WHO clinical stage, Adherence, Functional status, ART regiment, other comorbid condition (OCC), and Opportunistic infection(OIs) other than TB were considered as independent variables that were expected to be the predictors for the time to viral load suppression among HIV positive patients.

### Kaplan–Meier estimator

The figure in Kaplan–Meier survival curves shows if the pattern of one survivorship function lying above another, which means the group defined by the upper curve had more favorable survival experience than the group defined by the lower curve. However, it is unlikely to detect a difference when survival curves cross each other.

### Cox proportional hazards model

In Cox proportional hazards **(**PHs) regression model, the measure of effect is the hazard rate, which is the risk of failure (i.e., the risk of suffering to suppressed viral load count), given that the participant has survived up to a specific time. According to PHs assumption, the effect of a covariate is to increase or decrease the hazard by a proportionate amount which does not depend on any time (t). These indicated that the baseline hazard is a function of t but does not involve any predictor (X’s), whereas the exponential expression involves the *X*’*s* but does not involve t. Then, the Cox PHs model can be formulated as;

1 $${\text{h}}\left({\text{t}},\mathrm{ X},\beta \right)={h}_{o }\left(t\right)*Exp({\sum }_{1}^{p}{{\varvec{\beta}}}_{{\varvec{i}}}{{\varvec{X}}}_{{\varvec{i}}})={ h}_{o }\left(t\right)*Exp\left({{\varvec{\beta}}}^{\mathbf{^{\prime}}}{\varvec{X}}\right)$$

where ${\text{h}}\left({\text{t}},\mathrm{ X},\beta \right)$ the hazard function, ${h}_{o }\left(t\right)$ is the unspecified baseline hazard function and represents the hazards when all of the independent variables ${X}_{1} , {X}_{2 },{X}_{3 }{\cdots \cdots X}_{P}$ included in the model equal to zero. ${{\varvec{\beta}}}^{\boldsymbol{^{\prime}}}={(\beta }_{1} ,{\beta }_{2, } {\beta }_{3} \dots \dots {\beta }_{P } )$ is a vector of unknown regression coefficients and ${X=X}_{1} , {X}_{2 },{X}_{3 }{\cdots \cdots X}_{P}$ is a vector of time-fixed covariates. This hazard function is the product of two functions: The function ${h}_{o }\left(t\right)$ characterizes how the hazard function changes as a function of survival time and also the other function, $expx{\prime}\beta$ characterizes how the hazard function changes as a function of subject covariates^15^.

### Checking assumption of proportional hazard (PH) model

Before proceeding with other survival statistical model, it is obligatory to check PH assumptions. PHs assumption can be tested by using GLOBAL test. Small p-values indicate PH assumption holds rejected under the null hypothesis.

### Accelerated failure time model

Accelerated Failure Time** (**AFT) model is an alternative modeling frameworks to the PH model for the analysis of survival time data when the PH assumptions don’t satisfies^16^. Under AFT models we measure the direct effect of the explanatory variables on the survival time instead of hazard. This characteristic allows for an easier interpretation of the results because the parameters measure the effect correspondent covariates on the mean survival time^15^. Suppose ${x}_{1},{x}_{2},{x}_{3}\dots \dots \dots \dots ..{x}_{p}$ explanatory variables recorded for each individual in a study. According to the general AFT model, the hazard function of the ${i}$th individual is then,

2 $${h}_{i}\left(t\right)={h}_{o}\left(\frac{t}{{e}^{\mathrm{\eta i}}}\right){e}^{-\mathrm{\eta i}}$$

where ${h}_{o}({\text{t}})$ is the baseline hazard function and $\eta i$ is the acceleration factor that is a ratio of survival times corresponding to any fixed value of ${h}_{i}\left(t\right)$.

The acceleration factor is given according to the formula where $\eta i={\beta }_{1}{x}_{1i}+{\beta }_{2}{x}_{2i}+\cdots \cdots \cdots {\beta }_{P}{x}_{pi}$ is the linear component of the model in which ${x}_{ji}$ is the value of ${j}^{th}$ explanatory variable ${X}_{j}$ for the ${i}^{th}$ individual. According to the relationship of survival function and hazard function, the survival function for an individual with *p* covariates is given by: ${S}_{i}(t)$=${S}_{o}(t/{e}^{\mathrm{\eta i}})$. The factor ${\eta }_{i}$ is telling us how a change in covariate values changes the time scale from the baseline time scale. Depends on the nature of the data different forms AFT models considered like: Weibull, log-logistic and log normal AFT model.

### Model selection criteria

Model Selection criteria are crucial to compare the AIC and BIC values in order to determine which survival model are more appropriate for HIV patient’s data. Finally, the model with the lowest AIC and BIC values among these data is appropriate model.

### Univariable variable selection

First selected each covariates at 25% level of significance and statistically significant covariates were selected for multivariable analysis. The data were analyzed with statistical R software version 4.1.3.

### Model diagnostics checking

The plots of Cox‐Snell residuals can also be used in the graphical valuation of the adequacy of a fitted model. Thus, the plot of Cox-Snell residuals should give a straight line with unit slope, zero intercept and indicated the fitted model is good.

### Ethics approval and consent to participate

A statement to confirm that all methods were performed in accordance with the ethical standards as laid down in the declaration of Helsinki. Hence, an informed consent was waived by Bahir Dar University research technical and ethical review board with Ref.no Stat-S/166/2022 G.C by reason of retrospective nature of the study. The study was approved by Bahir Dar University research technical and ethical review board.

## Results

Among the 378 active participants in this study, 131 (66.1%) had a baseline CD4 cell count greater than 200 cells/mm^3^, and 184 (73.6%) of them had suppressed viral load during the study period. Similarly, 255 (67.5%) of the baseline viral load counts less than 10,000 copies per milliliter, of which 230 (90.2%) had suppressed viral load. On the other hand, among the 198 (52.4%) individuals whose hemoglobin level greater than 11g/dl had 146 (73.7%) suppressed viral load. Regarding weight and body mass index (BMI) of patients, 220 (58.2%) and 172(45.5%), had weight less than 50 kg and BMI greater than or equals to 25 kg/m^2^ respectively, of which 81.4% and 85.1% had suppressed viral load. In terms of the participants' functional status, 260 (68.8%), 86 (22.8%), and 70 (8.5%) were ambulatory, working, and bedridden respectively. 139 (36.8%) of the study subjects had clinical stage II, of which 101 (72.7%) experienced viral load suppression. Among the 145(38.4%) patients treated with 1e ART medication, 86.9% had suppressed viral load. 78.3% of patients with good treatment adherence had viral load suppressed. On the other hand, patients with-out other comorbid condition (OCC) and opportunistic infections (OIs) other than TB had 79.9% and 83.0% suppressed viral load (Table 1).

**Table 1:** Baseline clinical features of adult HIV patients.

Variables	Categories	Survival status		Total (%)
		Not suppressed (%)	Suppressed (%)	
CD4 cell	< 200	18 (14.1)	110 (85.9)	128 (33.9)
	≥ 200	66 (26.4)	184 (73.6)	131 (66.1)
Baseline viral load	≥ 10,000	59 (48.0)	64 (52.0)	123 (32.5)
	< 10,000	25 (9.8)	230 (90.2)	255 (67.5)
Hemoglobin level	< 11	32 (17.8)	148 (82.2)	180 (47.6)
	≥ 11	52 (26.3)	146 (73.7)	198 (52.4)
Wight	< 50	43 (27.3)	115 (72.8)	158 (41.8)
	≥ 50	41 (18.6)	179 (81.4)	220 (58.2)
BMI	< 18.5	48 (27.9)	127 (72.1)	172 (45.5)
	18.5–24.9	23 (19.3)	96 (80.7)	119 (31.5)
	≥ 25	13 (14.9)	74 (85.1)	87 (23.0)
WHO clinical stage	Stage-I	25 (18.4)	111 (81.6)	136 (36.0)
	Stage-II	38 (27.3)	101 (72.7)	139 (36.8)
	Stage-III	12 (15.0)	68 (85.0)	80 (21.2)
	Stage-IV	9 (39.1)	14 (60.9)	23 (6.0)
Adherence	Poor	22 (68.8)	10 (31.3)	32 (8.5)
	Fair	26 (52.0)	24 (48.0)	50 (13.2)
	Good	36 (12.2)	260 (87.8)	296 (78.3)
Functional status	Working	18 (6.9)	242 (93.1)	260 (68.8)
	Ambulatory	41 (47.7)	45 (52.3)	86 (22.8)
	Bedridden	25 (79.1)	7 (21.9)	70 (8.5)
ART regiment	1d	18 (25.7)	52 (74.3)	70 (18.5)
	1c	16 (16.0)	84 (84.0)	100 (26.5)
	1e	19 (13.1)	126 (86.9)	145 (38.4)
	Other	31 (49.2)	32 (50.8)	63 (16.7)
OCC	No	55 (18.3)	245 (81.7)	300 (79.4)
	Yes	29 (37.2)	49 (62.8)	78 (20.6)
OIs other than TB	No	49 (17.0)	240 (83.0)	289 (76.5)
	Yes	54 (60.7)	35 (39.3)	89 (23.5)

Over half of the patients in this study (65.3%) were female, and 71.1% of them suppressed viral load during the study period. In terms of patient ages in years, 188 (49.7%) patients were primarily between the age groups between 25 and 34 years; only 81.4% of these participants had a suppressed viral load. Additionally, 33.6% patients lived in rural areas and 66.4% in urban areas. Likewise, 106 (75.2%) of the 141 (45.2%) married participants had suppressed VL. In terms of religion, 275 (72.8%) had adherents to an orthodox faith, and of them, more than half (82.5%) suppressed viral load. In terms of religious status, 275 (72.8%) were followers of an orthodox religion, and of these, more than half (82.5%) had a suppressed viral load. Out of the total, 132 (34.9%) had secondary education, and 80.3% of them reduced the viral load. Similarly, 310 (82.0%) and 247 (65.3%) of the patients disclosed their status to family members and substance users, respectively. Of these, more than half (82.0%) and 65.3% suppressed the viral load (Table 2).

**Table 2:** Demographic characteristic of adult HIV patients.

Variables	Categories	Survival status		Total (%)
		Not suppressed (%)	Suppressed (%)	
Gender	Female	38 (45.2)	209 (71.1)	247 (65.3)
	Male	46 (54.8)	85 (28.9)	131 (34.7)
Age	15–24	18 (48.6)	19 (51.4)	37 (9.8)
	25–34	35 (18.6)	153 (81.4)	188(49.7)
	35–44	20 (25.0)	60 (75.0)	80 (21.2)
	> 44	11 (15.1)	62 (84.9)	73 (19.3)
Residence	Rural	46 (36.2)	81 (63.8)	127 (33.6)
	Urban	38 (15.1)	213 (84.9)	251 (66.4)
Marital status	Single	9 (17.6)	42 (82.4)	51 (13.5)
	Marriage	35 (24.8)	106 (75.2)	141 (45.2)
	Divorced	16 (18.2)	72 (81.8)	88 (23.3)
	Widowed	13 (29.5)	31 (70.5)	44 (11.6)
	Separated	11 (20.4)	43 (97.7)	44 (11.6)
Religion	Muslim	15 (40.5)	22 (59.5)	37 (9.8)
	Orthodox	48 (17.5)	227 (82.5)	275 (72.8)
	Other	21 (31.8)	45 (68.2)	66 (17.5)
Level of education	Non-educated	20 (22.7)	68 (77.3)	88 (23.3)
	Primary	16 (15.2)	89 (84.8)	105 (27.8)
	Secondary	26 (19.7)	106 (80.3)	132 (34.9)
	Tertiary	22 (41.5)	31 (58.5)	53 (14.0)
Disclosure status	No	39 (57.4)	29 (42.6)	68 (18.0)
	Yes	45 (14.5)	265 (85.5)	310 (82.0)
Substance use	No	29 (11.7)	218 (88.3)	247 (65.3)
	Yes	55 (42.0)	76 (58.0)	131 (34.7)

### Survival status of adult HIV positive patients

Out of 378 adult HIV patients, 84 (22.2%) were censored and 294 (77.8%) had a suppressed viral load. The mean and median survival times, which were 34.9 and 36.0 months respectively, had standard errors of 0.8 and 1.6 (Table 3).

**Table 3:** Adult HIV patients Mean, Median, and overall Survival status, SE indicated standard error of the estimate and CI indicated Confidence interval of the estimate.

Mean				Median				Survival status	
Estimate	SE	95% CI		Estimate	SE	95% CI		Censored (%)	No of event (%)
		Lower Bound	Upper Bound			Lower Bound	Upper Bound		
34.880	0.854	33.207	36.553	36.0	1.591	32.882	39.118	294 (77.8)	84 (22.2)

### Kaplan Meier estimate of the survival function

**Figure 2:**
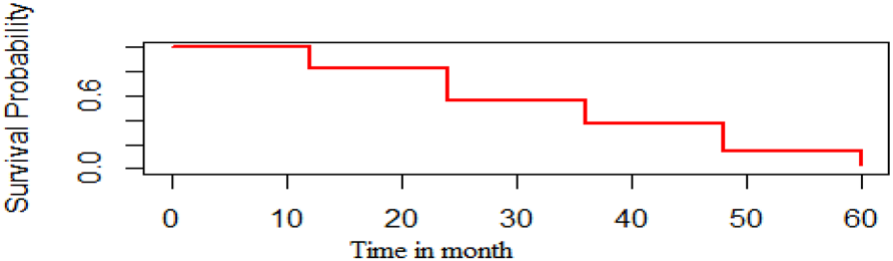
Adult HIV positive patients Kaplan–Meier plot survival function.

According to Kaplan–Meier survival curves, the overall survivor function in this study declined monotonically as the patient's time in month increased. I.e. as the patient's time in month increased, the adult HIV positive patients' survival probability demolishes (Fig. 2).

### Kaplan–Meier Curve for baseline hemoglobin level, CD4 cell count, adherence, and OCC

**Figure 3:**
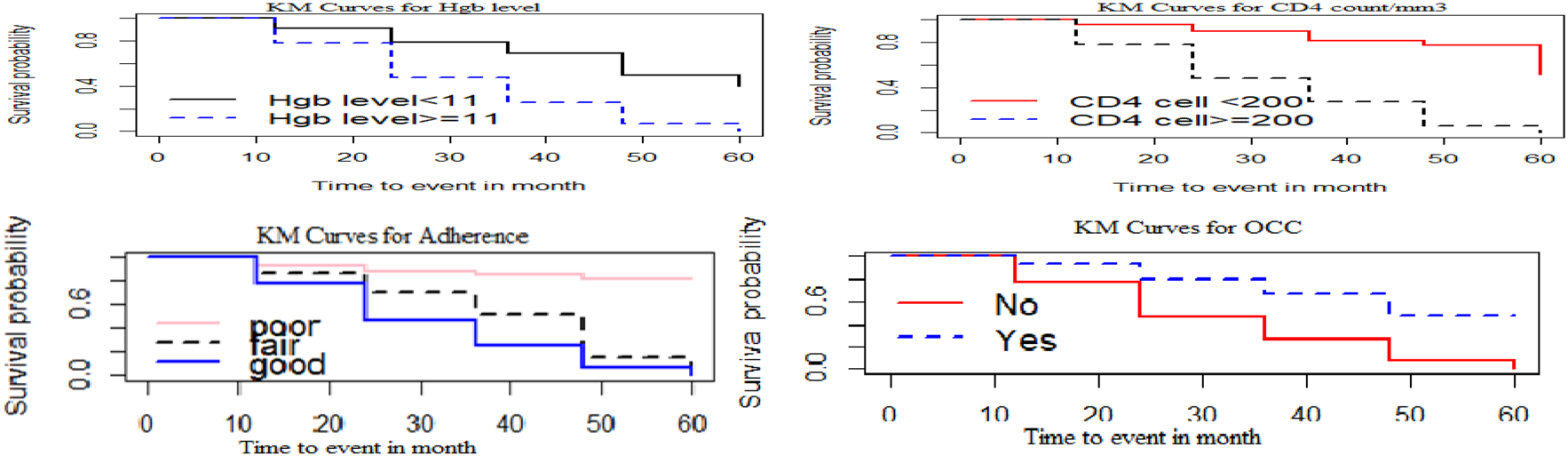
Kaplan–Meier Survival curve for baseline hemoglobin level, CD4 cell count, adherence, and OCC.

Patients with hemoglobin levels less than 11 g/dL and CD4 counts less than 200 cells/mm^3^ had a longer survival time than those patients with than hemoglobin level ≥ 11 g/dl and CD4 cell count ≥ 200 cells/mm^3^. Similarly, patients with poor adherence to treatment and OCC had longer survival time than those in the corresponding categories. i.e. The Kaplan–Meier survival curve suggested that the above curves are lower risk of suppression than their counterparts (Fig. 3).

### Evaluation of proportion hazard assumption

First we want to check the proportional hazard assumption before trying to any AFT model. The goodness of fit test gives a significant global p-value is less than 5% (Table 4). Since, in this study the global null hypothesis suggested that the proportionality assumption is rejected, and leads to PH model is unsuitable. As a result, we have used the AFT model, to analyze the survival time to viral load suppression for adult HIV positive patients.

**Table 4:** Goodness of fit testing for PH assumption.

Covariates	Chi-square	Degree of freedom	p value	Covariates	Chi-square	Degree of freedom	p value
Age	4.16502	3	0.244	CD4 cell count	0.00564	1	0.940
Sex	0.07900	1	0.779	Weight	0.40265	1	0.526
Education	2.04025	3	0.564	BMI	0.34343	2	0.842
Religion	0.31235	2	0.855	Adherence	6.68983	2	0.035
Residence	5.83226	1	0.016	WHO stage	6.17087	3	0.104
Marital status	7.16030	4	0.128	ART Regiment	6.21025	3	0.102
Disclosure	2.63970	1	0.104	Functional status	1.98903	2	0.370
Substance use	0.07967	1	0.778	OCC	0.61760	1	0.432
Baseline viral load	0.81079	1	0.368	OIs	0.05159	1	0.820
Hemoglobin level	0.10544	1	0.745	Global test	50.74383	34	0.032

### Accelerated failure time (AFT) model fitting

In the univariable AFT model analysis related to time to viral load suppression, the covariates age in years, educational status, residence, disclosure, baseline viral load, hemoglobin level, CD4 cell count, weight, body mass index, adherence, WHO clinical stage, ART regiment, OCC, and substance use are statistically significant. In contrast, gender, religion, marital status, and functional status are not significant at a 25% level of significance. A multivariable AFT mode is used to reanalyze all of the chosen univariable covariates.

### Comparison of AFT models

According to the model comparison criterion, the Weibull accelerated failure time model AIC and BIC values were minimal. Thus, the Weibull accelerated failure time model is a better baseline distribution than the others model for the data set of adult HIV positive patients (Table 5).

**Table 5:** Model comparisons for accelerated failure time models.

Model	Exponential	Weibull	Log-logistic	Lognormal
AIC	2739.63	2486.308	2513.839	2501.188
BIC	2877.352	2627.964	2655.496	2642.844

### Results of multivariable Weibull accelerated failure time model

Table 6 displays the multivariable Weibull accelerated failure time model results. The survival time to viral load suppression of HIV positive patients between the ages of 25 and 34 years was decelerated by a factor of 0.774 (φ = 0.774, 95% CI 0.602–0.793). The survival time to viral load suppression of patients with primary education was 0.931 (φ = 0.931, 95% CI 0.809–0.964) less than that of non-educators. Disclosure status of patients is a significant predictor of time to viral load suppression, and patients disclose the disease to family members are at a higher risk of viral load suppression as compared to non-disclosure with an acceleration factor of 1.093 (φ = 1.093, 95% CI 1.001–1.4570). In terms of initial viral load count, those with < 10,000 copies/mL had a higher survival time to viral load suppression for HIV positive with an acceleration factor of 1.153 (φ = 1.153, 95% CI 1.015–1.309) than those with Viral load count ≥ 10,000 copies/mL. Patients who had a hemoglobin level of ≥ 11 g/dL and a CD4 count of ≥ 200 cells/mm^3^ were substantially linked to poor patient survival, with a factor of 1.147 (φ = 1.147, 95% CI 1.019–1.290) and 1.145 (1.145) (φ = 1.145, 95% CI 1.028–1.275). In a similar way, patients who weight more than 11 kg and had a BMI between 18.5 and 24.9 kg/m^2^ were found to be substantially more likely to have poor HIV patient survival, with corresponding acceleration factors of 1.151 (φ = 1.151, 95% CI 1.033–1.275), and 1.143 (φ = 1.143, 95% CI 1.007–1.296). Considering survival time to viral load suppression of a fair treatment adherence group of patients was significantly associated with poor survival as compared to poor treatment adherence. This is revealed by a time ratio of less than one (0.867) (φ = 0.867, 95% CI 0.778–0.967) indicating a larger survival time and lower risk of viral load suppression. On the other hand, a time ratio more than one (1.2) (φ = 1.200, 95% CI 1.046–1.377) indicating a lower survival time and higher risk of viral load suppression. Individuals with OCC had a considerably longer survival time, with a roughly one-times (φ = 0.821, 95% CI 0.720–0.936) lower survival time viral load suppression. HIV patients' time to viral load suppression is significantly predicted by substance use, and those who use drugs have a somewhat lower chance of suppression than those who do not use 0.876 (φ = 0.876, 95% CI 0.773–0.993) (Table 6).

**Table 6:** Results of multivariable Weibull AFT model, $\lambda$ = 2.4606, AIC = 2462.162, BIC = 2613.70, *Statistically significant at 5% level of significance; *β* coefficient, *φ = exp(β)* an acceleration factor, *CI* confidence interval for φ, *S.E.* standard error, *Ref* reference, $\lambda$ scale parameter.

Variables	Categories	β	S.E.	p-value	φ	95% CI for φ	
						Lower CI	Upper CI
Age (Ref: 15–24)	25–34	− 0.2556	0.0118	0.030*	0.774	0.602	0.793
	35–44	− 0.2208	0.1288	0.086	0.802	0.623	1.032
	> 44	− 0.1986	0.1265	0.116	0.819	0.639	1.051
Education (Ref: non-education)	Primary	− 0.0717	0.0178	0.018*	0.931	0.809	0.964
	Secondary	− 0.0431	0.0681	0.527	0.958	0.838	1.095
	Tertiary	0.1510	0.0968	0.101	1.163	0.962	1.406
Residence (Ref: rural)	Urban	− 0.0457	0.0597	0.444	0.955	0.849	1.074
Disclosure (Ref: no)	Yes	0.1887	0.0958	0.035*	1.093	1.001	1.457
Viral load (Ref: ≥ 10,000)	< 10,000	0.1423	0.0649	0.028*	1.153	1.015	1.309
Hgb level g/dl (Ref: < 11)	≥ 11	0.1357	0.0549	0.016*	1.145	1.028	1.275
CD4 count/mm^3^ (Ref: < 200)	≥ 200	0.1372	0.0600	0.001*	1.147	1.019	1.290
Weight (Ref: < 50)	≥ 50 kg	0.1409	0.0552	0.011*	1.151	1.033	1.275
BMI (Ref: < 18.5)	18.5–24.9	0.1333	0.0644	0.038*	1.143	1.007	1.296
	≥ 25	0.1056	0.0698	0.130	1.111	0.969	1.274
adherence (Ref: poor)	Fair	0.1422	0.0556	0.001*	1.867	1.778	1.967
	Good	0.1826	0.0701	0.027*	1.200	1.046	1.377
WHO (Ref: stage-I)	Stage-II	0.0330	0.1623	0.103	1.031	0.752	1.421
	Stage-III	0.0720	0.0686	0.294	1.074	0.939	1.229
	Stage-IV	− 0.0805	0.0129	0.007*	0.923	0.899	0.946
ART regiment (Ref:1d)	1c	− 0.0393	0.0801	0.624	0.961	0.822	1.125
	1e	− 0.0942	0.0789	0.233	0.910	0.779	1.062
	Other	− 0.0690	0.1025	0.501	0.933	0.763	1.141
	Bedridden	0.2995	0.1654	0.070	1.349	0.976	1.866
OCC (Ref: no)	Yes	− 0.1971	0.0667	0.005*	0.821	0.720	0.936
OIs (Ref: no)	Yes	− 0.0256	0.0167	0.201	0.975	0.943	1.007
Substance use (Ref: no)	Yes	− 0.1322	0.0638	0.038*	0.876	0.773	0.993

Source: UGCSH 30th March 2017–30th March 2022.

### Overall goodness of fit

**Figure 4:**
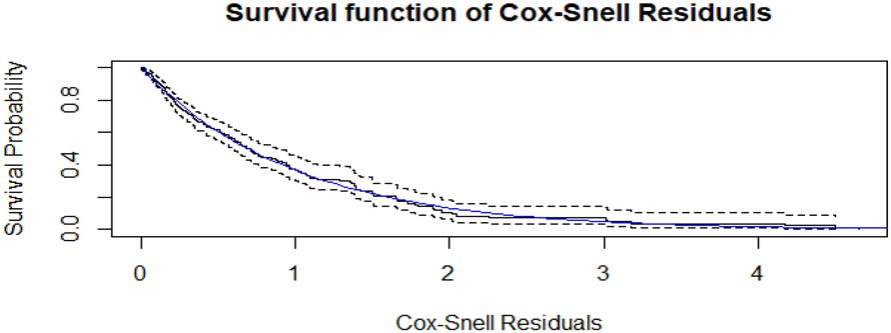
Cox–Snell residual plots for the Weibull AFT model of adult HIV patients.

The blue line in the Cox–Snell residual plot displayed the model diagnosis with 95% confidence interval (CI) for the Kaplan–Meier estimate of the Cox-Snell. The blue line represents the survival function of the unit exponential distribution, while the black solid line is the Kaplan–Meier estimate of the survival function of the residuals (with the dotted lines corresponding to the 95% pointwise confidence intervals). This finding showed that the adult HIV positive patient's data suited the Weibull AFT model effectively (Fig. 4).

## Discussion

In this retrospective analysis, 77.8% of HIV patients suppressed their viral load while receiving antiretroviral therapy. This aligns with findings from earlier research conducted in Cambodia, which revealed 76.8%^17^, 89% found in Uganda^18^, and 93% in Vietnam^19^. Patients in ages between 25 and 34 years were significant predictors for survival time to viral load suppression. Previous study not supports this result^11^. These discrepancies could be due to differences in study areas, follow-up periods, and/or hospital facility existence.

Results of the current study revealed that educational level is a significant predictor of time to viral load suppression. This could be primary educators has better chance of understanding the treatment follow up and possible to virally suppressed as compared to non-educators. This study is inconsistent with a study done at Harare City^13^ and Ethiopia^10^ which states that primary educators were not independent risk factors for survival time.

The survival time to viral load suppression was higher for HIV patients under disclosed the disease to family member. This could be attributable to the maximizing immunity level of patients at expressed the status of the disease to family member. This study is support with former study conducted in Harare City^13^, in their results indicated that study participants expressed the status of the disease to family member had a nearly fourfold increased risk of suppression compared to non-disclosure patients. However, this findings were inconsistent with another study conducted in Arba Minch, Ethiopia^11^, which found that patients under the category of disclosed the disease to family member were not independent predictors to viral load suppression. These differences could be due to sample size, study periods, study area and methods of data analysis.

This study also illustrated that baseline viral load is a significant predictor to time to viral load suppression. Patients with low baseline viral load (< 10,000 copies/mL) experienced the higher viral load suppression than those with high baseline viral load ((≥ 10,000 copies/mL).The result of this study is supported by the findings from other studies^11^. These findings are not shocking due to the fact that the higher plasma viral load means the larger HIV reservoirs; therefore it takes longer survival time to achieve viral load suppression. Similarly, results of the current study revealed that the time to viral load suppression of patients whose CD4 count ≥ 200 cells/mm^3^ was shorter than those who have < 200 cells/mm^3^ CD4 count. This finding agrees the findings of the study conducted in different settings^11,19,20^. This result predicted that higher CD4 cell counts usually correlate with low viral load concentration and therefore patients take shorter time to viral load suppression.

Furthermore, weight of patients has also an effect on the duration of viral load suppression. The effect of weight on time to viral load suppression might be due to the fact that, weights ≥ 50 kg has a significant reduction in serious bacterial infections and mortality, this improvement correspond to a substantial success in early viral load suppression.

BMI category 18.5–24.9 kg/m^2^ had increased risk of viral load suppression. The result of this study is consistent with a study done by^10^. This study also showed that good and fair treatment adherence of patients was onefold higher to experience early viral load suppression as compared to those who have poor treatment adherence. As a matter of fact that adherence is the key, and potentially modifiable predictors associated with time to viral load suppression. The idea of these study is supported by a study done at Uganda^18^.

In the current investigation also shown WHO clinical stage is predictors for time to viral load suppression. Previous study had confirmed these result, in their findings shown that HIV patients with WHO clinical stage-IV have a viral load suppression than clinical stage-I^10^. This could be because individuals with advanced WHO clinical stages were more likely to get additional opportunistic infections like TB. However, this study is inconsistent with a study done at Southern Brazil^21^ and democratic republic of Congo^22^.

Results of the current study revealed that HIV positive individual users are slightly lower risk than non-users. On the other hand substance drug user patients had poor health status and resulting lower viral load suppression than non-users. This study is inconsistent with the literature^10^. Similarly, this study also showed that patient with OCC was lower to experience early viral load suppression as compared to patient’s with-out OCC.

## Conclusion

In this current study Weibull AFT model was better than the other AFT models. The findings of this study showed that the predictors that were statistically associated with higher time to viral load suppression were patients disclosed the disease to others, baseline virial load count less than 10,000 copies/mL, hemoglobin level ≥ 11 g/dL, CD4 cell count ≥ 200/mm^3^, weight ≥ 50 kg, body mass index between 18.5 and 24.9 kg/m^2^, fair and good adherence. The other important predictors were age in year between 25 and 34 years, primary education level, advanced WHO clinical stage, patients with OCC, and substance users statistically associated with lower time to viral load suppression. Then, near intensive care of adult patients’ whose ages between 25 and 34 years, primary educational level, advanced WHO clinical stage, patients with OCC, and substance users can help them in order to enhance their quality of life and extend their lifespan.

## Limitation of the study

This study was based on retrospective cohort study design, the data obtained from adult HIV positive patients chart. However, some important socio-demographic and clinical predictors like nutritional status, income status, homeownership, and other hematological parameters like eosinophil, neutrophil, and basophil counts, were not available in participant charts.

## Data Availability

The data used in the current investigation is available from the corresponding author and can be attached upon request. The data accessed in the current investigation complied with relevant data protection and privacy regulations.

## Abbreviations

AFT: Accelerated failure time model
AIC: Akaike information criteria
AIDS: Acquired immune deficiency syndrome
ART: Antiretroviral therapy
BIC: Bayesian information criterion
BMI: Body Mass Index
CD4: Cluster differentiation 4
G.C: Gorgerin calendar
Hgb: Hemoglobin
HIV: Human immune deficiency virus
OIs: Opportunistic infections
PHs: Proportional hazards
UGSCH: University of Gondar Comprehensive Specialized Hospital
VL: Viral load
WHO: World Health Organization

## Code availability

All codes organized in this study are available from the corresponding author on reasonable request.
